# Sodium-Glucose Cotransporter 2 (SGLT-2) Inhibitors: Delving Into the Potential Benefits of Cardiorenal Protection Beyond the Treatment of Type-2 Diabetes Mellitus

**DOI:** 10.7759/cureus.16868

**Published:** 2021-08-04

**Authors:** Natasha Srinivas, Mubashira K Sarnaik, Srimy Modi, Yasaswi Pisipati, Sarayoo Vaidya, Naqvi Syed Gaggatur, Aliya H Sange, Ibrahim Sange

**Affiliations:** 1 Research, BGS Global Institute of Medical Sciences, Bangalore, IND; 2 Internal Medicine, MS Ramaiah Medical College, Bangalore, IND; 3 Research, KJ Somaiya Medical College, Mumbai, IND; 4 Internal Medicine, MS Ramaiah Medical College, Bengaluru, IND; 5 Research, Dubai Medical College, Dubai, ARE; 6 Research, California Institute of Behavioral Neurosciences & Psychology, Fairfield, USA; 7 Medicine, KJ Somaiya Medical College, Mumbai, IND

**Keywords:** sglt2 inhibitors, diabetes mellitus, cardiovascular disease, heart failure, chronic kidney disease, diabetic nephropathy, pharmacokinetics, pharmacodynamics

## Abstract

Diabetes mellitus is a leading cause of morbidity and mortality and a significant risk factor for the early onset of chronic kidney disease and heart disease. Hyperglycemia and insulin resistance are key factors that play a role in the pathogenesis of type 2 diabetes. Renal glucose reabsorption is a critical component of glycemic regulation. Sodium-glucose cotransporter 2 (SGLT2) inhibitors, commonly known as gliflozins, lower blood sugar levels by inhibiting glucose absorption in the proximal tubule of the kidney. SGLT2 inhibitors are currently used primarily as antidiabetic medications; however, their advantages go well beyond just glycemic control. This article has reviewed the mechanisms behind cardiac and renal involvement in type 2 diabetes and their inseparable interconnections. This article has also discussed the pharmacokinetic and pharmacodynamic profile of different SGLT2 inhibitors available in the market. Finally, this review has provided a perspective on the outcome trials, which provide evidence supporting a potential benefit of SGLT2 inhibitors in reducing cardiovascular and renal risks and possible mechanisms that mediate the renal and cardiovascular protection conferred.

## Introduction and background

Diabetes mellitus is a serious and widespread chronic disease caused by an intricate interaction between genes and the environment, as well as other risk factors, including obesity and a sedentary way of life [1]. It is believed to affect more than 415 million adults worldwide and with increasing prevalence, it is estimated that it will affect more than 640 million adults by 2040 [2-3]. Diabetes is characterized by chronically elevated blood glucose levels as a result of an inability of the beta cells of the pancreas to generate enough insulin or inefficient insulin use by body cells [4]. Diabetes mellitus type 2 (T2DM) is significantly linked to the development of cardiovascular disease (CVD) and heart failure (HF), with diabetic patients being hospitalized four times more often for HF than non-diabetic patients [5-6]. The formation of advanced glycation end products (AGE), which act on and activate a particular receptor, receptor for advanced glycation endproducts (RAGE), is a significant mechanism by which hyperglycemia accounts for unfavorable clinical outcomes in T2DM [7]. AGEs can cause cardiac fibrosis and myocardial stiffness, leading to heart failure with preserved ejection fraction (HFpEF), in addition to microangiopathy and endothelial dysfunction [7]. Diabetes is also linked to an elevated risk of adverse renal outcomes, with diabetic kidney disease being one of the major contributing causes to end-stage renal disease [8]. T2DM contributes to kidney disease by producing glomerular hyperfiltration, mesangial expansion, and the build-up of extracellular matrix, ultimately leading to mesangiolysis and glomerular fibrosis [7]. Sodium-glucose cotransporter 2 (SGLT2) inhibitors constitute a novel class of oral hypoglycemic agents (OHAs) that have been authorized for use in the treatment of T2DM [9]. SGLT2 is responsible for the reabsorption of 97% of the entire glucose filtered by the kidney [9]. SGLT2 inhibitors suppress renal glucose reabsorption, thus enhancing urinary glucose excretion and effectively lowering blood glucose levels [9]. SGLT2 inhibitors further decrease body weight and visceral adipose tissue and improve the parameters of blood pressure, lipid profile, and serum uric acid level, all of which are closely correlated with metabolic syndrome [10]. Recent cardiovascular outcome trials (CVOTs) with SGLT2 inhibitors have shown to boost cardiovascular and renal outcomes in patients both with T2DM and without T2DM, with the American Diabetes Association (ADA) and The European Association for the Study of Diabetes (EASD) recommending them as a core component of T2DM therapy [11]. At present, commercially available SGLT2 inhibitors include ipragliflozin, dapagliflozin, canagliflozin, empagliflozin, luseogliflozin, and tofogliflozin, which have been approved for use as antihyperglycemic agents in patients with T2DM [12].

The objectives of this review article are to (1) Discuss the mechanism and pathophysiological involvement of the heart & the kidneys in T2DM and explain the inextricable interactions between them; (2) Review the pharmacokinetics & pharmacodynamics of SGLT2 inhibitors; (3) Summarize the cardiovascular and renal benefits of SGLT2 inhibitors in T2DM beyond glycemic control.

## Review

Diabetic heart disease

The key factor in the development of T2DM is insulin resistance, which causes insulin hypersecretion [7]. With the progression of the disease, the pancreas’ insulin output, albeit higher than normal, becomes insufficient to prevent hyperglycemia, indicating the presence of T2DM [7]. Increased insulin resistance, while contributing to hyperglycemia, also enhances the absorption and metabolism of free fatty acids by cardiomyocytes, leading to the accumulation of triglycerides and lipotoxicity, which leads to an impairment in cardiac contractility [13]. Another important mechanism in diabetes is the formation of AGEs, which act on their receptors, leading to the formation of reactive oxygen species (ROS), thus promoting inflammation in the myocardium and microcirculation [7]. Inflammation and oxidative stress lead to cardiac myocyte apoptosis and mitochondrial dysfunction, thereby diminishing adenosine triphosphate (ATP) generation, which may further lead to reduced calcium uptake by the sarcoplasmic reticulum, interfering with contraction [14]. AGEs can also lead to cardiac fibrosis, which causes myocardial stiffness, leading to diastolic heart failure. As described by Seferovic and Paulus, both phenotypes of heart failure in diabetes are secondary to molecular alterations in myocardial structure and function, which lead to diabetic cardiomyopathy (DCM) [7,15]. Diabetes increases the risk of morbidity and mortality, secondary to both systolic and diastolic heart failure [16]. T2DM is also characterized by the formation of extensive lipid-containing atherosclerotic plaques, which are vulnerable to rupture [17]. These, along with the prothrombotic state due to an increase in coagulation factors and impaired fibrinolysis, increase the likelihood of coronary thrombosis and myocardial infarction (MI) [17].

Diabetic kidney disease (DKD)

Chronic kidney disease (CKD), clinically characterized by impaired renal function or elevated urinary excretion of albumin or both, affects approximately half of all patients with T2DM [18]. A microvascular disorder, DKD is associated with numerous morphological changes in multiple kidney compartments, with the earliest being thickening of the glomerular basement membrane [19]. Thickening of tubular and capillary basement membrane follows suit [19]. Loss of endothelial fenestration, expansion of mesangial matrix, and podocyte loss with effacement of foot processes are some of the other glomerular alterations seen in DKD. With the progression of diabetes, segmental mesangiolysis is seen along with the development of microaneurysms and Kimmelstein-Wilson nodules [20]. Subendothelial deposits of plasma proteins cause hyaline arteriolosclerosis and can cause luminal compromise. In later stages of the disease, interstitial changes and glomerulopathy consolidate to form segmental and global sclerosis [21].

**Figure 1:**
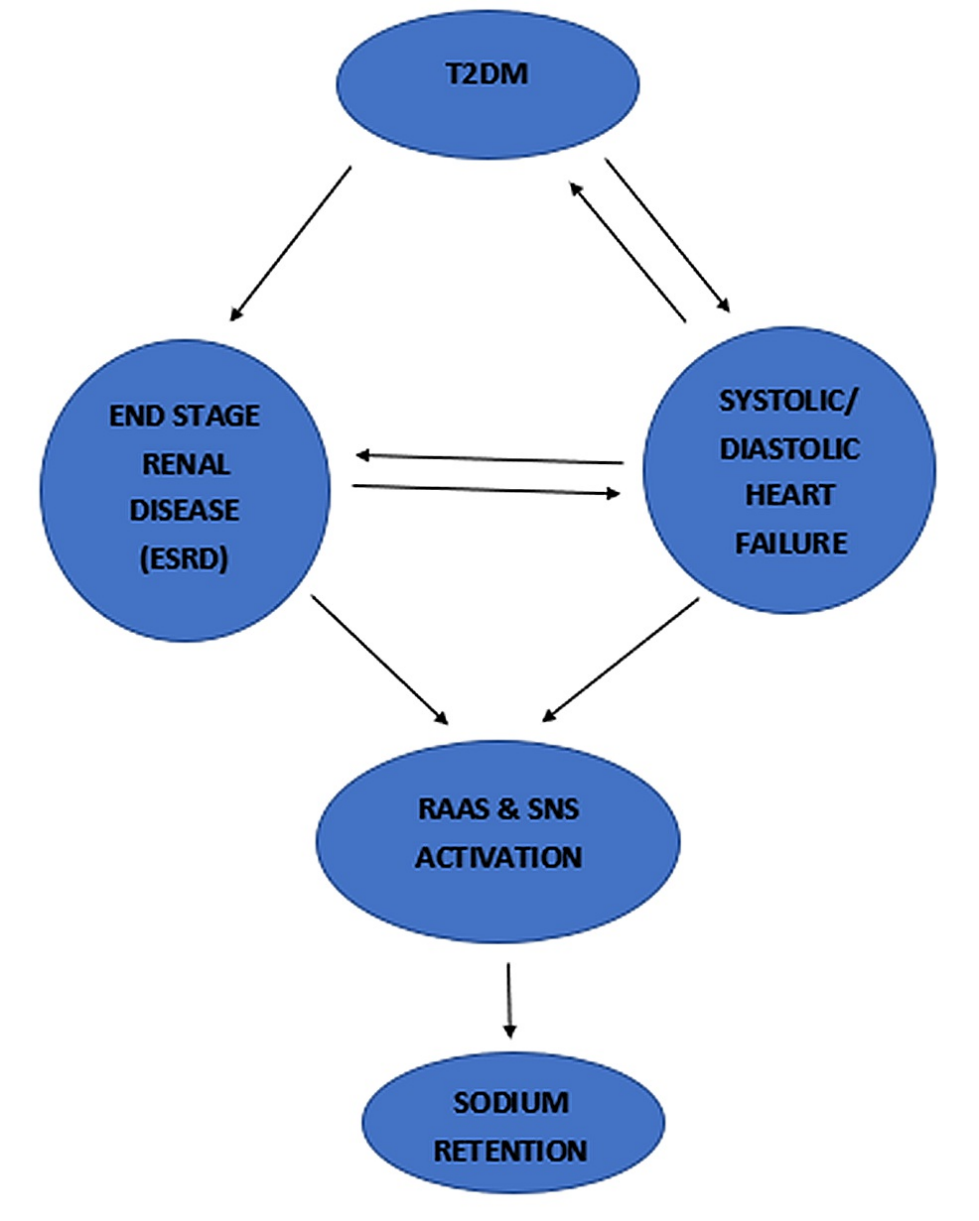
Involvement and interactions between the heart and kidneys in diabetes mellitus ESRD- End-Stage Renal Disease; RAAS- Renin Angiotensin Aldosterone System; SNS- Sympathetic Nervous System

Vicious circles in diabetes involving the heart and the kidney are shown in Figure 1.

SGLT2 inhibitors: pharmacodynamics

The glomerulus filters glucose readily and thereafter, it is reabsorbed in the proximal convoluted tubule (PCT). The maximum renal glucose reabsorptive capacity (TmG) in humans is about 375 mg per minute, with men having somewhat higher TmG than women [22]. The rate at which glucose is filtered in normal glucose-tolerant persons (~180g per day or ~125mg per minute) is significantly lower than the TmG, meaning all filtered glucose is reabsorbed with none present in the urine. However, in individuals with poorly managed diabetes mellitus, the filtered glucose load surpasses the reabsorptive capacity of the kidney, causing glycosuria. In healthy individuals, no glycosuria appears until blood glucose levels exceed 180 mg/dl, referred to as the threshold for glycosuria [22]. Because glucose is a polar molecule that cannot permeate through the walls of the proximal convoluted tubule (PCT), it is reabsorbed by secondary active transport with the help of two sodium-glucose cotransporters present on the apical membrane of the PCT, namely, SGLT2 and sodium-glucose cotransporter 1 (SGLT1) [23]. The pharmacodynamic effect of SGLT2 inhibitors is inducing glycosuria by one of the three mechanisms - by decreasing the TmG, lowering the threshold for glucose reabsorption, or by increasing splay (difference between ‘theoretical’ and ‘actual’ threshold) [23]. Dapagliflozin, for example, reduced TmG by 56% in well-controlled T2DM patients (from 420 mg/min to 184 mg/min) [24]. Despite normal blood glucose levels, non-diabetic individuals also have glycosuria; this glycosuria in the presence of normal glycemia is explained by a significant drop in the glucose resorption threshold from 180 mg/dL to 40-80 mg/dL [24].

SGLT2 inhibitors: pharmacokinetics

Dapagliflozin

The first SGLT2 inhibitor to be approved in the world, the pharmacokinetics of dapagliflozin has been extensively studied. It is available both as monotherapy and combination therapy along with metformin and gliptins. Following oral administration, it is rapidly and widely absorbed from the gastrointestinal (GI) tract with an oral bioavailability of 78% [25]. Bioavailability is not significantly altered with the intake of a high-fat meal, allowing for administration irrespective of meals [25]. It reaches maximum plasma levels (Tmax) 1-1.5 hours after intake and has a plasma half-life (T1/2) of 13 hours, making it suitable for once-a-day dosing [25]. Uridine-5'-diphosphate-glucuronosyltransferase 1A9 (UGT1A9) catalyzes the conversion of dapagliflozin to inert glucuronide conjugates, predominantly dapagliflozin 3-O-glucuronide, which are then excreted in the urine [26].

Canagliflozin

The first SGLT2 inhibitor to be approved for use in the United States, like dapagliflozin, it is administered once a day before the first meal; however, it is slightly less selective for SGLT-2 (250-fold selectivity as compared to 1200 times with dapagliflozin) [27-28]. The bioavailability is around 65%, with a T1/2 varying between 11- 13 hours [27-28].

Empagliflozin

Approved for use in 2014, empagliflozin has the highest selectivity for SGLT2 among SGLT2 inhibitors [23]. Taken once daily in the morning, recommended dosing is 10 mg, which can be titrated up to 25 mg [29]. Studies show an oral bioavailability varying between 60% and 75%, with a Tmax of one hour and T1/2 of about 13 hours. Glucuronidation is a major metabolic pathway for empagliflozin, with metabolites eliminated by both fecal and renal routes [29].

Sotagliflozin

The first dual SGLT1/SGLT2 inhibitor to reach phase 3 trials, it only has a modest affinity for SGLT2 over SGLT1 (about 20-fold) [30]. It has been demonstrated that taking it right before breakfast maximizes its effectiveness [30]. It has a rapid onset of action with Tmax of three hours and T1/2 varying between 13 and 20 hours in patients with preserved renal function [30]. Despite its predominant clearance by the kidney, in patients with renal illness, plasma elimination of sotagliflozin is not significantly affected [30].

Other SGLT2 inhibitors and their pharmacokinetic parameters are listed in Table 1.

**Table 1:** Pharmacokinetics of different SGLT-2 inhibitors SGLT-2- Sodium-Glucose Cotransporters Type 2; ESRD- End-Stage Renal Disease; NA- Not Available; FDA- Food and Drug Administration

SGLT-2 inhibitor	Trade name	Route/ Dosing (mg)	Bioavailability (%)	Time to peak action/ Tmax (hours)	Plasma protein binding (%)	Half-life (hours)	Metabolism/elimination	Contraindications
Dapagliflozin [25,26]	Farxiga (USA)	Oral/5,10	78	1-2	91	13	Extensive hepatic glucuronidation/renal & fecal	Hypersensitivity, ESRD/severe renal impairment & pregnancy
Canagliflozin [27,28]	Invokana (USA)	Oral/100,300	65	1-2	98	11-13	Extensive O-glucuronidation/fecal & renal	Hypersensitivity, ESRD/severe renal impairment & pregnancy
Empagliflozin [29]	Jardiance (USA)	Oral/10,25	60-75	1	86	13	Extensive glucuronidation & oxidation to a lesser degree/ renal & fecal	Hypersensitivity, severe renal impairment, ESRD & pregnancy
Sotagliflozin [30]	(Under investigation, not FDA approved)	Oral/200, 400	-	3	-	13-20	Excretion mostly renal	-
Ipragliflozin [31]	Suglat (Japan)	Oral/25, 50	90	1-2	-	10-13	Metabolism by glucuronidation/fecal	Hypersensitivity, severe renal impairment, ESRD & pregnancy
Luseogliflozin [32]	Lusefi (Japan)	Oral/2.5, 5	NA	1-2	-	10-12	NA	NA
Tofogliflozin [33]	Apleway, Deberza (Japan)	Oral/20	97.5	0.5-1.5	83	5-6	Predominant oxidative metabolism/renal & fecal	Hypersensitivity, severe renal impairment, ESRD & pregnancy
Ertugliflozin [34]	Steglatro (USA)	Oral/5	70-90	0.5-1.5	-	11-17	Extensive glucuronidation/ renal & fecal	Hypersensitivity, severe renal impairment, ESRD & pregnancy

Cardiorenal benefits of SGLT2 inhibitors

The Food and Drug Administration (FDA) mandated cardiovascular safety trials for novel antidiabetic treatments in 2008 while the European Medicines Agency mandated them in 2012. The goal of these large-scale randomized clinical trials was to demonstrate the non-inferiority of SGLT2 inhibitors for major adverse cardiac events (MACE) when compared to traditional antidiabetic medications, with superiority as a secondary outcome [35]. In general, these trials enrolled participants with high cardiovascular risk [35].

Table 2 lists the main clinical trials of SGLT2 inhibitors assessing cardiovascular and renal outcomes, with some of them conducted in patients also having diabetic kidney disease (DKD) and heart failure with reduced ejection fraction (HFrEF).

**Table 2:** SGLT2 inhibitor trials in adults with cardiovascular and renal disease CANVAS- Canagliflozin Cardiovascular Assessment Study; CREDENCE- Evaluation of the Effects of Canagliflozin on Renal & Cardiovascular Outcomes in Participants with Diabetic Nephropathy; CV- Cardiovascular; DECLARE-TIMI 58: Dapagliflozin Effect on Cardiovascular Events- Thrombolysis In Myocardial Infarction 58; eGFR- Estimated Glomerular Filtration Rate; EMPA-KIDNEY- Study of Heart and Kidney Protection With Empagliflozin; EMPA-REG OUTCOME- Empagliflozin Cardiovascular Outcome Event Trial in Type 2 Diabetes Mellitus patients; ESKD- End-Stage Kidney Disease; HHF- Hospitalization for Heart Failure; MACE- Major Adverse Cardiac Events; MI- Myocardial Infarction; NA- Not Available; RRT- Renal Replacement Therapy; SCr- Serum Creatinine; T2DM- Type 2 Diabetes Mellitus; UACR- Urinary Albumin to Creatinine Ratio

	EMPA-REG OUTCOME [36]	CANVAS PROGRAM [37]	DECLARE-TIMI 58 [2]	CREDENCE [38]	EMPA-KIDNEY [39]
No. of patients	7,020	10,142	17,160	4,401	5,000
Intervention	Empagliflozin 10 mg or 25 mg versus placebo	Canagliflozin 100 mg or 300 mg versus placebo	Dapagliflozin 10 mg versus placebo	Canagliflozin 100 mg versus placebo	Empagliflozin versus placebo
Patient Population	T2DM & established CV disease	T2DM & established CV disease or >/= 2 CV risk factors	T2DM & established CV disease or risk factors for CV disease	CKD + T2DM	CKD +/- T2DM
eGFR (ml/min/1.73 m^2^)	>/= 30	>/= 30	>/= 60	30-89	>/= 20 - <45 or >/= 45 - <90 with UACR >/= 200 mg/g
Mean follow-up (years)	3.1	3.6	4.2	2.6	NA
Primary Outcome	Composite of CV death, non-fatal MI, or non-fatal stroke	Composite of CV death, non-fatal MI, or non-fatal stroke	Composite of CV death, non-fatal MI, or ischemic stroke hospitalization for HF	New ESKD or doubling of serum creatinine level or renal or cardiovascular death	CV death or kidney disease progression
Secondary Outcomes (CV)	CV death, non-fatal MI, non-fatal stroke, hospitalization for unstable angina; CV death; All-cause mortality; Fatal or non-fatal MI; Fatal or non-fatal stroke HHF	CV death; All-cause mortality; Fatal or non-fatal MI; Fatal or non-fatal stroke; HHF	CV death; All-cause mortality; Fatal or non-fatal MI; Fatal or non-fatal stroke; HHF	All-cause mortality; CV death; MACE; HHF	All-cause mortality; CV death
Secondary Outcomes (Renal)	Doubling of SCr with GFR less than or = 45, RRT or renal death; ESKD; Doubling of SCr; Worsening nephropathy; Progression to macroalbuminuria	Doubling of SCr, ESKD, or death from renal causes; ESKD; Doubling of SCr; Progression to macroalbuminuria	Renal composite: 40% decrease in eGFR to <60ml/min/1.73 m^2^, ESKD or renal death	Renal composite: Doubling of SCr, ESKD, or renal death; Doubling of SCr; ESKD; Renal death	CV death or ESKD; Renal disease progression

Cardiovascular Protective Mechanisms

In light of the results of cardiovascular outcome trials, which reported reduced hospitalizations and a decrease in major adverse cardiac events (MACE), several theories have been proposed to account for these observations. In general, these are attributed to the following four hypotheses [40].

1. Hemodynamic effects, including natriuresis and a reduction in effective circulating volume and blood pressure, thereby reducing both preload and afterload [40].

2. Increased ketogenesis, which in turn can be used for adenosine triphosphate (ATP) generation [41]. Inhibition of SGLT2 decreases glucose oxidation in both baseline and insulin-stimulated settings while increasing fat oxidation [41]. Increased renal ketogenesis may also be responsible for an increase in hematocrit seen in SGLT2 inhibition, which in turn increases oxygen delivery to vascular beds susceptible to hypoxia, like renal medulla and myocardium [41].

3. Reduced myocardial sodium-hydrogen exchange, which is linked to a reduction in cardiac fibrosis, remodeling, and systolic dysfunction [42]. SGLT2 inhibitors block the sodium-hydrogen exchanger in the heart, lowering sodium and calcium levels in the cytoplasm of cardiomyocytes while increasing mitochondrial calcium [42]. This enhances the excitation-contraction coupling and antioxidant capacity of the mitochondria [42].

4. Suppression of sympathetic nervous system (SNS), thus decreasing the risk of arrhythmia-related sudden cardiac death [43].

Renal Protective Mechanisms

As evidenced by the EMPA-REG outcome trial, treatment with empagliflozin significantly decreased renal events such as progression to macroglobinuria, increasing serum creatinine levels, the need for renal replacement therapy, and death due to renal failure by 39% [36]. The following mechanisms are associated with improved renal outcomes in diabetics taking SGLT2 inhibitors:

1. Restoration of tubuloglomerular feedback: The actions of SGLT2 inhibitors in the proximal convoluted tubule (PCT) results in increased sodium chloride delivery to the macula densa, which results in the release of vasoactive substances like adenosine [12]. Adenosine, through its receptor, causes vasoconstriction of the afferent arteriole, thereby reducing glomerular pressure and hyperfiltration [12].

2. Reducing tubular workload and hypoxia: Energy and adenosine triphosphate (ATP) is required for the resorption of electrolytes and organic solutes in the proximal tubule. The proximal tubule is responsible for the majority of oxygen consumption in the kidney [44]. Diabetics have enhanced proximal tubular glucose reabsorption through SGLT2 due to increased intraluminal glucose caused by hyperglycemia and glomerular hyperfiltration [45]. This increased reabsorption creates an increased demand for oxygen by the PCT, rendering it comparatively hypoxic [46]. Tubular hypoxia is a crucial factor in the progression of diabetic kidney disease (DKD) [46]. SGLT2 inhibitors lower tubular workload and ameliorate hypoxia in the proximal tubule by decreasing salt and glucose reabsorption. Empagliflozin has also been found to enhance mitochondrial function and autophagy by decreasing renal tubular mitochondria fragmentation via AMPK (AMP-activated protein kinase) activation [47].

3. Diuretic and natriuretic effect: SGLT2 inhibitors cause natriuresis and glucosuria, which leads to diuresis [12]. SGLT2 inhibitors mobilize fluid preferentially from the interstitial compartment rather than the intravascular compartment [48]. Along with reducing blood pressure and the risk for heart failure, this helps reduce the interstitial fluid in the kidney, which may alleviate cortical and medullary hypoxia.

4. Anti-inflammatory and antifibrotic effect: Nuclear factor-B (NFB), interleukin 6 (IL-6), monocyte chemoattractant protein 1 (MCP-1), and other factors linked to inflammation and tissue fibrosis are reduced by SGLT2 inhibitors [49]. In clinical studies of SGLT2 inhibitors in individuals with T2DM, similar effects on urine IL-6 and MCP-1, as well as serum tumor necrosis factor receptor 1 (TNFR1) and IL-6, were reported [49]. SGLT2 inhibitors are also said to reduce serum uric acid levels in diabetics, thus reducing the risk of kidney inflammation [50].

**Figure 2:**
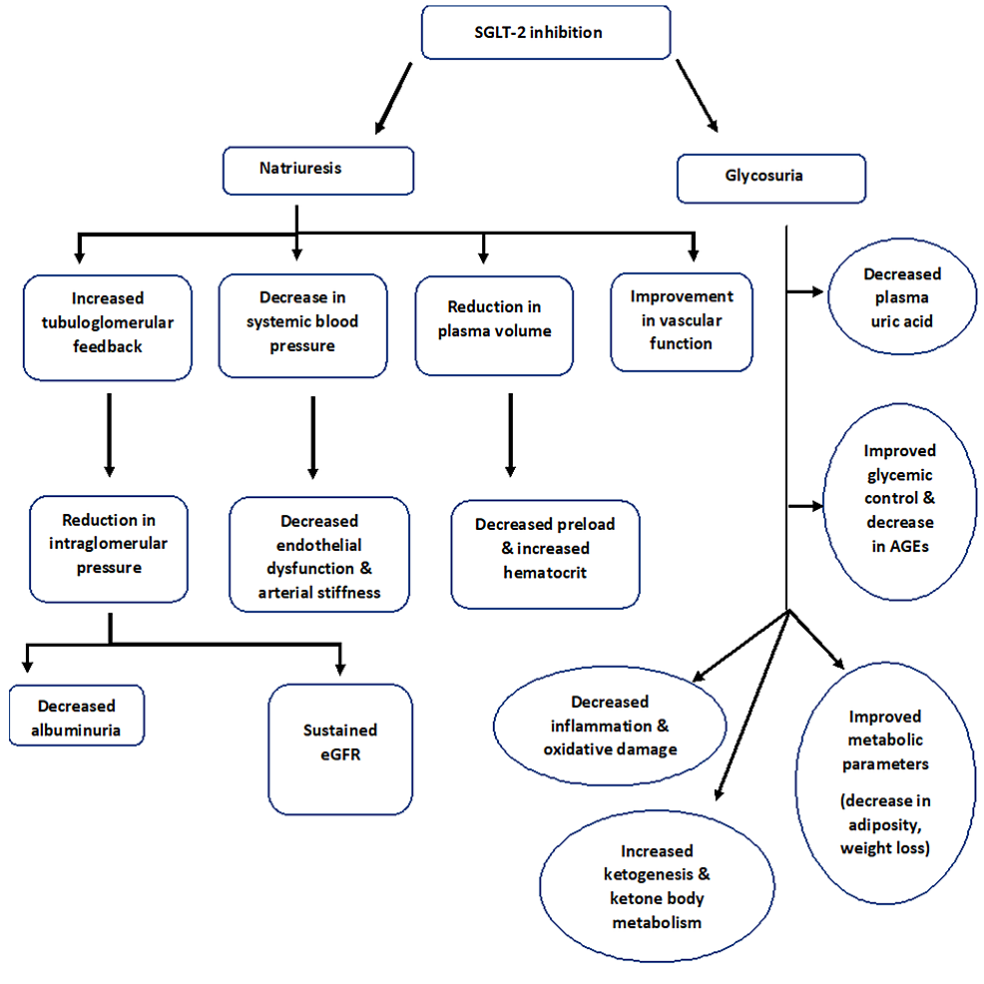
Overview of effects of SGLT-2 inhibitors leading to cardiorenal protection SGLT-2- Sodium-Glucose Co-Transporter 2; AGE- Advanced Glycation End Product; HbA1C- Hemoglobin A1C; eGFR- Estimated Glomerular Filtration Rate

An overview of the effects of SGLT2 inhibitors is shown in Figure 2.

Limitations

Diabetes mellitus is a complex disease with a multifaceted causal network, with treatment comprising lifestyle modifications, weight loss, and pharmacological drug therapy, depending on the therapeutic objective. This article particularly focuses on SGLT2 inhibitors and does not consider other etiological factors and therapies used in the treatment of type 2 diabetic patients.

Whether SGLT2 inhibitors confer a nephroprotective benefit even at very low estimated glomerular filtration rate (eGFR) levels is something that is yet to be determined, with studies using hard renal endpoints and parameters still underway.

## Conclusions

SGLT2 inhibitors are a novel class of antidiabetic drugs that have shown great potential in treating cardiovascular and renal sequelae in patients with type 2 diabetes. Aside from their well-established glucose-lowering properties, emerging data suggest they may have a role in cellular stress, metabolic homeostasis, and inflammation. As evidenced from the cardiovascular outcome trials, SGLT2 inhibitors have been shown to lower the incidence of atherosclerotic disease, including myocardial infarction and non-fatal stroke, and decrease heart failure-related sequelae and cardiovascular death. In major clinical trials charting renal outcomes, they were also shown to reduce the progression of diabetic kidney disease and the requirement for renal replacement therapy. Patients at various stages of cardiac, renal, and metabolic diseases should be included in future trials to ensure that the existing evidence is generalizable. Results of ongoing and future randomized controlled trials will undoubtedly broaden the clinical indications across a wide range of patient populations, enabling us to discover and unlock more benefits of SGLT2 inhibitors.
